# Convolutional Neural Network Bootstrapped by Dynamic Segmentation and Stigmergy-Based Encoding for Real-Time Human Activity Recognition in Smart Homes

**DOI:** 10.3390/s23041969

**Published:** 2023-02-09

**Authors:** Houda Najeh, Christophe Lohr, Benoit Leduc

**Affiliations:** 1 Lab-STICC, IMT Atlantique, 29280 Brest, France; 2 Delta Dore Company, Le Vieux Chêne, 35270 Bonnemain, France

**Keywords:** real-time human activity recognition, convolutional neural network, directed weighted network, overlapping activities

## Abstract

Recently, deep learning (DL) approaches have been extensively employed to recognize human activities in smart buildings, which greatly broaden the scope of applications in this field. Convolutional neural networks (CNN), well known for feature extraction and activity classification, have been applied for estimating human activities. However, most CNN-based techniques usually focus on divided sequences associated to activities, since many real-world employments require information about human activities in real time. In this work, an online human activity recognition (HAR) framework on streaming sensor is proposed. The methodology incorporates real-time dynamic segmentation, stigmergy-based encoding, and classification with a CNN2D. Dynamic segmentation decides if two succeeding events belong to the same activity segment or not. Then, because a CNN2D requires a multi-dimensional format in input, stigmergic track encoding is adopted to build encoded features in a multi-dimensional format. It adopts the directed weighted network (DWN) that takes into account the human spatio-temporal tracks with a requirement of overlapping activities. It represents a matrix that describes an activity segment. Once the DWN for each activity segment is determined, a CNN2D with a DWN in input is adopted to classify activities. The proposed approach is applied to a real case study: the “Aruba” dataset from the CASAS database.

## 1. Introduction

In the literature, different simulation tools support engineers to optimize building design [1]. It is proved that the behaviors of occupants are important to explain the discrepancy between the actual and the simulated energy consumption [2]. Understanding the behaviors of occupants might reduce this gap. Recently, many researchers have focused on modeling occupants’ activities in order to improve simulation performance [3,4].

An important number of research works show that HAR technologies are very powerful in pre-segmented activity sequences [5,6]. However, the implementation of some real-time applications are necessary for continuous HAR on progressive sensor data.

As part of reactive smart home technologies, HAR on streaming data allows for knowledge about which activities the occupant is currently performing. Let’s consider the case of piloting rolling shutters: The motorization of roller shutters simplifies daily actions, however, an opening of the rolling shutter in the morning at 8:00 a.m. when the occupant is sleeping impacts the occupant’s perception. In this case, it is possible to program the opening and closing of the shutters according not only to the climatic conditions but also to the activities of occupants in real time.

The recognition of occupant activities depends not only on data segmentation but also on collected features from installed sensors which can discover relationships between these features with human practices. The use of sensors comes from the assumption that when occupants perform some activities, they interact with the surroundings, affecting environmental conditions (CO_2_ concentration, air temperature, air humidity, etc.) and consuming electricity, for example, when using appliances (microwave, computer, TV, etc.).

Smart home ambient sensors record the actions of occupants in their dwellings. These recordings are the event logs that catch the daily activities in the house. The event log is transformed into a univariate or multivariate time series. As in many areas of HAR, a standard approach is to first segment the data. In [7], different strategies of segmentation (or windowing) are studied and compared. These techniques include sensor event window (SEW) [8], time window (TW) [9], and fuzzy time window (FTW) [10]. These methods are easy to implement, however, the main related challenge is to judge whether or not two sequential events belong in the same activity segment. Therefore, in this paper we used a real-time dynamic sensor event segmentation methodology that incorporates the calculation of spacial correlation between events. This can eliminate the placement of sensor events with weakly spatial correlation in the same sliding activity segment.

Following real-time segmentation, classification of the segments of activities is important for the task of HAR. Feature extraction is an intermediary step between segmentation and classification. The activities may overlap between different functional areas, and there is no standard way to accomplish an activity by occupants. Therefore, the feature extraction should take into account the information related to spatio-temporal characteristics of activity (i.e., trajectory of occupant during activity in different areas). The main contribution of this paper is to propose an encoding architecture for HAR in smart home technology. Ants algorithm [11,12] is used to explicitly represent the extracted features.

This work proposed an online human activities’ recognition schema on streaming data from binary sensors. The methodology integrates three steps: (1) a dynamic segmentation method, (2) encoding, and (3) classification of activities using a deep learning technology (a CNN2D architecture) to test the efficiency of the proposed encoding technique. The dataset Aruba [13] is employed to evaluate our proposed methodology.

This paper is structured as follows: The related works are summarized in Section 2. The proposed methodology is detailed in Section 3. Section 4 investigates the case study and the experimental results. Finally, concluding remarks are given in Section 5.

## 2. Reported Works

A smart home is a home containing home automation devices such as sensors and actuators (presence sensors, thermometer, light control, shutters, etc.). It provides new services to occupants related to security, comfort, and energy management. In addition to these services, sensors also make it possible to predict the practices of the residents, determining the energy consumption by predicting the number of occupants per zone as well as their activities and routines. Occupant activities are defined as what a person or a group does or has done in a certain place for a certain time [14].

Currently, many scientists are working on modeling human activities in order to improve the performance of energy simulation tools. Initially, activity scenarios are programmed and defined for each heating control zone. These scenarios are determined based on standard conditions or statistical analysis of observations. However, these scenarios are reproducible for many households while their characteristics (members, type of building, professional, etc.) are different. Consequently, they are not suitable enough to model the activity of occupants in all their diversity.

The activity of an occupant can indeed be attached by the context in the dwelling, which provides a set of surrounding circumstances that can be used to characterize the situation of this activity [15,16]. For example, a family of four cooks differently than a family of one. A person can sleep longer on weekends than on weekdays.

Human activity recognition techniques in smart homes are pattern recognition algorithms. The existing techniques can be split into two categories: data-driven methods (DDM) and knowledge-driven methods (KDM). The first category uses data generated by the user to model and recognize the activity, and it is based on machine learning and data mining techniques. The second category uses rule-based tests, prior knowledge of the domain, and logical reasoning by an expert.

KDMs are based on the observation that most activities and routines of work and daily life take place in a relatively circumscribed place and time. References [17,18] proposed context and activity ontologies for explicit domain modeling. The limits of the proposed technique include the requirement of a complete knowledge of the domain to elaborate an activity model, and its weakness in handling uncertainty and adaptability to new settings.

Bayesian network (BN) is a method to predict the activities of occupants based both on dataset and on expert knowledge. The contribution of [14] proposed a general method that takes into account a particular context to estimate the human activities. Specifically, an activity consequences-based Bayesian network is built based on measurement data collected from sensors (temperature, CO_2_, and energy consumption of electrical appliances, etc.) and knowledge of a residential building. The results are evaluated with an F1-score above 85%. Reference [19] built a BN to predict the usage of ovens in cooking activity based on the expert structure of effect nodes (duration, energy) and causal nodes (hour, type of day, month). Bayesian network is flexible and understandable to occupants. It leads to advantages for the residents to validate and explain the model. However, all BN-based methods focus on simple activities such as presence, actions of doors/windows, and usage of appliances.

The DDM approaches for human activity recognition require labeled data on which the learning algorithm is trained. After the training step, the algorithm is then able to classify the unknown data. The DDA’s strength is its probabilistic modeling capacity. These models are able to handle noisy, uncertain, and incomplete sensor data. They can also capture domain heuristics, e.g., some activities are more likely than others. They do not require a predefined domain knowledge. However, they require much data, and producing quality training datasets is expensive to meet mandatory criteria: data of a significant size, in a usable form, the need for tools and manpower (qualified and rigorous) to label data, and control steps to avoid bias and errors in the dataset.

In the literature, there is an important number of learning techniques used for recognizing human activities, such as support vector machines (SVM) [20], hidden Markov models (HMM) [21,22], naive Bayes [23], dynamic Bayes nets [21], and nearest neighbor [24]. These methods show potential in HAR tasks, and they have made great progress in this field of research. However, they are usually carried out in a hand-crafted way dependent upon occupant expertise and domain knowledge.

In [25], several classification techniques such as hidden Markov model, multi-layer perceptron, and decision trees are evaluated. They reported superior performance of LSTM, stochastic gradient descent of linear SVM, and SVM. In [14], a consequences-based graphical model for contextualized occupant activities estimation in connected buildings is studied. The obtained F1-score of three concerned activities (cooking breakfast, cooking lunch, and cooking dinner) are respectively 85 %, 80%, and 86 %.

In recent years, with the fast development of neural network technologies, the over limits have been surmounted to a sure degree. The most recently used techniques for human activity recognition are deep learning-based techniques [26]. As an example, [27] used three different artificial neural network (ANN) approaches to recognize human activities such as sleeping, bed to toilet, and bathing.

In the literature, the most used NN architectures to recognize human activities are convolutional neural network (CNN) and recurrent neural network (RNN) with its variations, such as LSTM. For example, in [10], an LSTM is combined with a fuzzy time window (FTW) to process the real-time HAR with an accuracy rating less than 96%. Reference [28] used a CNN2D as a classifier. To perform the feature extraction task, they turned activity sequences into binary images where the y-axis of the image represents the sensors grouped by zones. However, this work is limited to binary sensor use and there is a need to test the efficiency of this method on other types of sensors. Reference [29] proposed an HAR framework using a CNN architecture bootstrapped by a location-based stigmergy encoding technique for the emergent representation of daily activities. In [30,31], a CNN-LSTM architecture is investigated. It is a temporally and spatially deep architecture that ameliorates the performance of prediction and diminishes the complexity of the model. With these techniques, the features are automatically learned and a high level representation can be extracted in the deep layer. In this work, we extend the works mentioned above and employ a CNN2D deep learning architecture to accomplish the objective of real-time HAR.

Most models of deep learning use only pre-segmented datasets. However, human activities must be online tracked in many real-time scenarios. This requires the HAR schema to process pre-segmented sensor data and deal with streaming data. Related works in this research field are relatively scarce. For example, [32] investigated a dynamic segmentation technique of sensor events. The study used two scenarios—non-overlapping and overlapping time windows. Reference [33] proposed another dynamic segmentation technique for streaming data based on semantic analysis and statistical learning. In order to achieve the goal of dynamic adaptation, this method studied the input event sequence and selected the more appropriate time-window size. Reference [34] presented several methods of sliding windows that process streaming data. Five methods of fixed-size windowing with different weighting factors are proposed. The work presented in [35] proposed a method of dynamic segmentation on streaming data that integrated time correlation and event correlation and performed real-time HAR on streaming data.

In this paper, we used a real-time dynamic segmentation based on the calculation of event spatial correlation [35] with some rearrangement in the computations. For the case study, an apartment with labeled activities is employed to evaluate our framework.

## 3. Real-Time Human Activity Recognition Framework

This section describes the real-time HAR framework that integrates three steps: (1) Real-time dynamic segmentation method that determines the beginning and the end of each activity segment; (2) encoding; and (3) classification using a neural network architecture (CNN2D). These steps are preceded by a data pre-processing step.

**Figure 1:**
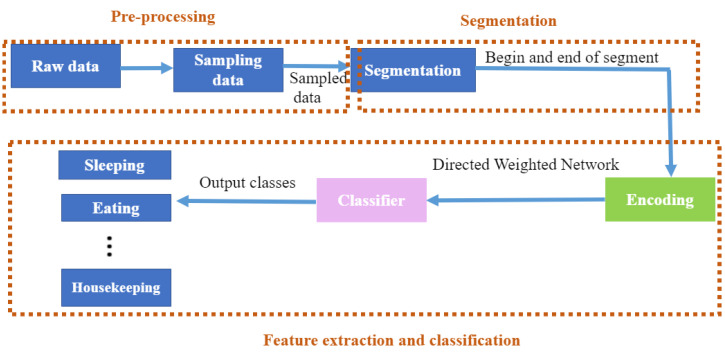
Real-time human activity recognition framework.

The whole process comprises two phases (offline and real-time phases). In the offline phase, the data are cleaned (i.e., delete duplicated data, correct the order of sensor activation traces which are not correctly temporarily ordered in the dataset) and sampled. The dynamic segmentation and the encoding step are the real-time phases, and the objectives of these steps are to establish the corresponding sliding window when a new sensor event occurs, and to encode this segment, respectively. These steps are followed by a multi-class recognition model, which classifies the ongoing activity. For each incoming event, the correlation between this event and the last event is calculated. If they are strongly correlated then they belong to the same segment, otherwise the last event is the start of a new segment. Once the beginning and end of each segment are determined, the segment is transformed to a representative trajectory of activity. This trajectory is calculated using a DWN which returns a matrix that describes the spatial-temporal traces of the occupant and that will be considered as input of a classifier which classifies the activity. The architecture of the proposed framework is shown in Figure 1.

Firstly, raw data from sensors are cleaned and sampled and then split using a real-time dynamic windowing (Section 3.1). The sliding windows are then encoded (Section 3.2), and finally classified by the CNN2D (Section 3.3).

### 3.1. Segmentation

In this paper, we used real-time dynamic segmentation on streaming data. It integrates the spacial correlation between events and determines whether or not two sequential sensor events belong to the same segment of activity. This ensures that events from very different zones will not be in the same window. This method is published in [35] and it allows for determining the beginning and the end of each segment when new events are inscribed.

Let $E=\{e_{1},e_{2},...,e_{n}\}$ represent a sequence of events, where $e_{i}$ represents the *i*th event. Each $e_{i}\in E$ contains a vector of information $<T_{i},s_{i},V_{i}>$ where $T_{i}$, $s_{i}$, and $V_{i}$ represent, respectively, time stamp of the *i*th event (date: year; month; day), time (hour; minute; second), sensor name of the *i*th event and sensor value of the *i*th event.

The concept of dynamic segmentation is the following: For each incoming event, the question that arises is whether the incoming event belongs to the current segment, or if it is the beginning of a new segment. To answer this question, we calculate the correlation between events using the Pearson product moment correlation (PMC) coefficient [31], which measures the linear correlation between two sensor events. In the following, the PMC coefficient is named $\rho_{X,Y}$ and it is calculated using Equation (1).

(1) $$\rho_{X,Y}=\frac{cov(X,Y)}{\sigma_{X}\sigma_{Y}}$$

cov(X,Y), $\sigma_{X}$, and $\sigma_{Y}$ represent the covariance of *X* and *Y*, the standard deviation of *X*, and the standard deviation of *Y*, respectively. Three cases could be highlighted: (1) *X* and *Y* are positively correlated when $\rho_{X,Y}=1$; (2) *X* and *Y* are negatively correlated when $\rho_{X,Y}=-1$; and (3) *X* and *Y* are not correlated when $\rho_{X,Y}=0$.

**Figure 2:**
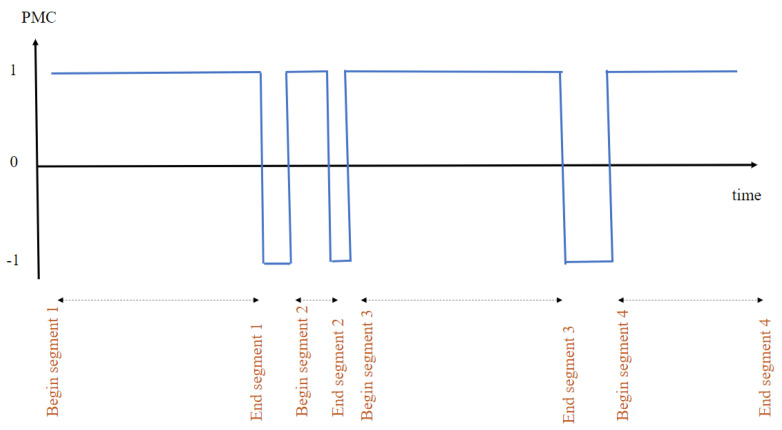
Real-time human activity recognition framework.

Figure 2 illustrates an example of identification of the beginning and the end of activities’ segments, i.e., how to process the real-time dynamic segmentation.

Once the correlation between events is determined for each incoming event, as long as the correlation is always equal to 1 it means that this event belongs to the current segment. As soon as the correlation is different to 1, the last sample corresponds to the end of the segment and as soon as it goes back to 1, this moment corresponds to the beginning of a new segment.

### 3.2. Encoding

In the previous section, we explained that the extraction of features is an important step for a human activity recognition system. From these features, a classifier attempts to identify patterns in order to associate the correct activity label. Therefore, human activity recognition can be reduced to a pattern recognition and classification problem. However, thanks to deep learning, in addition to patterns, these models are able to automatically extract features and finally perform the task of classification. Deep learning allows to consider models able to extract the features from raw data and to perform the classification task at the same time.

CNN models demonstrate potential in automatic feature extraction, pattern recognition, and classification. The CNN1D is used in many research works for real-time HAR and there is a lot of feedback on its classification capacity. The input of a CNN1D architecture is a sliding window of sensor event window (SEW) that contains encoded data. Depending on the SEW size and the activity length, some windows are padded with zero values to maintain a constant input length, a process called “zero padding”. Once all the activity sequences have been cut into smaller windows and annotated with the label of the activity from which it originates, they are distributed into subsets for training and testing data. In such research works as [6], it is demonstrated that “zero-padding” could drop the performance of classification because a CNN1D is unable to ignore the padding and might interpret it as useful data.

**Figure 3:**
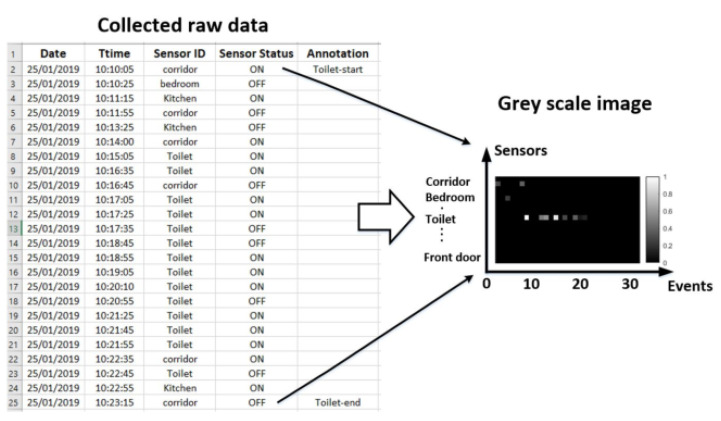
Gray scale image of the activity “bed to toilet”.

An activity is defined by a start time, an end time, and a set of events between the beginning and the end. The events involved in the activity are measured by binary sensors with two states (ON and OFF). The sensor ID represents the zone of the house in which the sensor is installed. Each timestamp represents the instant of sensor activation. The time difference between the instant of triggering of the last sensor event and that of the actual triggering sensor corresponds to a time delay. Therefore, in order to extract features and patterns in activities, authors in [28] used the concept of activity image; with a black background and white pixels that correspond respectively to each “OPEN” and “CLOSE” signal of the door sensors and "ON" and "OFF" signals of the motion detection sensors. Reference [36] used a grayscale image to represent a particular activity. Figure 3 shows a segment of “bed to toilet” activity as well the corresponding gray scale image, respectively. It is a binary image with a gray range of pixels that corresponds respectively to the “CLOSE” and “OPEN” signals of the door contact sensors and the “OFF” and “ON” signals of the motion sensors. The grayscale range corresponds to the duration of each sensor event. The two dimensions of the grayscale image are the X-axis (length) and the Y-axis (height), representing the time events of the activity and the sensor patterns of the activity, respectively.

However, these techniques are challenged by:

- A CNN requires images with the same size as input, whereas this is not always the case because the size of activity segments differs from one activity to another. Consequently, the activity images may not be the same size in terms of duration, and we come across the padding problem mentioned on the CNN1D. In this work, we will make the best use of these encoding techniques, not with pixels but with matrices.
- The extraction of features that contain information related to behavioral semantics as well as spatio-temporal characteristics is a challenge. Establishing an efficient representation to define the activity related information is also challenging because there are different manners of doing a type of activity and the overlapping activities between different zones in the home can lead to confusion between activities.

To solve the above issues, the adopted technique in this work is a stigmergy-based solution [11,12]. Stigmergy is an indirect mechanism of coordination between agents. The principle is that a trace left in the environment by an initial action stimulates a following action, by the same or a different agent. In this manner, consecutive actions suggest reinforcing each other, consequently leading to the automatic emergence of coherent systematic activity.

The most typical example of stigmergy is the collective movement of ants, coordinated by traces of pheromones, i.e., how anthills can automatically calculate efficient paths for workers. Ants deposit pheromones on the ground and are conditioned to follow the most fragrant path, which also happens to be the most densely traveled. Different deposits in the same place concentrate in intensity. Ants in a group can modify their behavior after detecting a special pheromone. The concentration of the pheromones decreases gradually over time. In connection with human activity recognition, we use stigmergy as a motif of information accumulation for spatio-temporal tracks of occupants. In fact, when sensors are activated by human movement, the correspondent marks with a time decay (i.e., a rate of reduction after a timestamp) will be released continuously in a computer-simulated space, which can accumulate the marks.

To distinguish information from different pheromone sources and hence infer the coarse stigmergic track of the occupant, a representation of the occupant’s trajectory as a directed weighted network (DWN) is used. This can give an indication of the placement of the occupant, as well as the concentration of pheromones when a sensor is activated. In this paper, a DWN and its related matrix are investigated to represent the stigmergic trajectory. We rely on the method presented in [31] to determine the DWN for each segment of activity that we have summarized here for more clarity, and we seek to extend this method by taking into account the overlapping of activities that take place in the same area.

**Figure 4:**
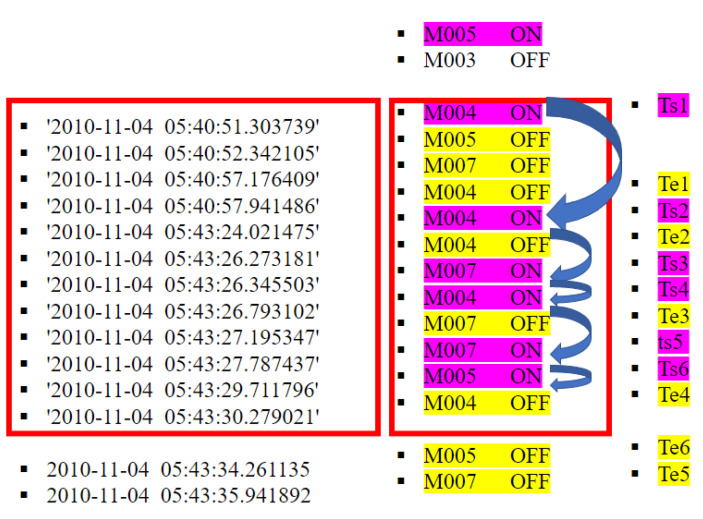
Segment of activity.

**Figure 5:**
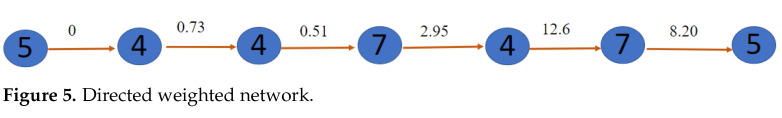
Directed weighted network.

**Figure 6:**
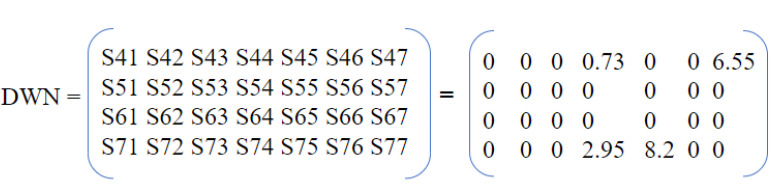
Resulting matrix.

Figure 4, Figure 5 and Figure 6 present an example of the process of DWN generation from a segmented sequence of data. The parameter ρ represents the volatility rate and its value is between 0 and 1. The selected window is framed by a red rectangle in Figure 4.

**The first step** of the design of a directed weighted network is to extract the triggered sensors, then their order of triggering (see Figure 4).

In this case, the triggered order of sensors is M005 →M004 →M004 →M007 →M004 →M007 →M005.

Because the sensors are activated by the movement of the occupant, the position of the triggered sensor is an indication of the location of the occupant at time *t*. Once the triggered order of sensors is determined, the DWN corresponds to that on Figure 5.

In this work, two types of loops are distinguished:

- A self loop between the first sensor triggered in the segment and the sensor triggered just before.
- The remaining directed stops.

For this loop type, we calculate the weights of each stop, i.e., duration of activation of sensors.

**The second step** of the proposed methodology is to determine the weight of each directed edge including self loops using Equation (2). To calculate the weights, we used the method proposed by [31] and we seek to extend this method by calculating the weights under constraint of non-overlapping activities. The weights represent the intensity of the pheromones deposited by the sensor that correspond to the right position of the pointer of the directed edge. In each period of time, a unit of pheromone is liberated in the corresponding position in the environment of the triggered sensor. The pheromone intensity decreases with a rate of volatility ρ after a time step (fixed to 1 s in this work). Without time volatility, the intensity of aggregated pheromones is equal to the activation duration $t_{e}-t_{s}$. When time volatility is introduced, the intensity of pheromones I is calculated using Equation (2), which describes the superposition of the newly generated pheromone and the volatilized old ones.

(2) $$I=\sum_{t=t_{s}}^{t_{e}-1}{\left(1-\rho \right)}^{T_{e}-t-1}=\frac{{\left(1-\rho \right)}^{T_{e}-t_{e}}-{\left(1-\rho \right)}^{T_{e}-t_{s}}}{\rho }$$

where *Te*, *te*, and *ts* represent the time of the last sensor event $e_{i}$ in the segmented window $W_{i}$, the trigger start time, and the trigger end time for each directed edge, respectively. The interpretation of start and end time of each sensor is the following: in this work, we privilege the activities on a short $\Delta_{t}$. The weights of the representative activity matrix are calculated on the duration $t_{e}-t_{s}$ where $t_{s}$ and $t_{e}$ represent the start time event (i.e., the history) and the actual time event, respectively.

The adjacent matrix of the DWN is represented by n rows and m columns. These rows and columns contain the sensors’ names involved in the activity segment. For each event, the number of rows is traversed with a counter *i* and the number of columns with a counter *j*. At a given instant, the transition $S_{i}$ to $S_{j}$ represents the transition between the last active sensor (whose index is *i*) and the current active sensor (whose index is *j*). For example, the transition from the sensor $S_{2}$ to $S_{3}$ represents the weight $S_{23}$ in the matrix, with the index 2 being the second row and the index 3 being the third column. If in the sensor triggering order, there is no transition $S_{i}$ →$S_{j}$ (for example $S_{3}$ →$S_{5}$) then $S_{35}$ will take the value 0 in the matrix. Let’s consider the example of transition M007 →M004 presented in Figure 6, where the end time $T_{e}$ is 05: 43: 26; the first trigger start time of M004 is 05: 43: 26; and the first trigger end time of M004 is 05: 43: 29. So, the directed edge $I_{7\to 4}=2.95$.

In the following, the directed weighted network and its adjacency matrix are collectively referred to as “DWN” and is used as input of the fine-grained classification algorithm. It gives information not only about the concentration of pheromones liberated when each sensor is triggered, but also about the placement of the occupant before the triggering of sensors. Also, it can distinguish different information about sources of pheromones and conclude a coarse stigmergic trajectory. Moreover, with this marker-based stigmergy paradigm, the intensity of the old pheromones decreases gradually over time due to the volatility, and the outcome on the context defining the last event is weakened gradually, while the effect of the new pheromones is greater relatively.

In this work, the corresponding matrix of DWN is calculated under constraint of non-overlapping activities (i.e., maximization of distance between activities occurred in the same functional area). To eliminate the overlapping between two activities (A1 and A2), we calculate the DWN for each activity segment, which return a matrix $M_{i}$ for the *i*th window of an activity segment using different values of ρ. It means that for each $\rho_{i}$, we obtain a distance $d_{i}\left(A1,12\right)$. So, the parameter ρ corresponds to the arguments of the maxima “argmax” of different distances.

$$\rho =Argmax_{\rho_{i}}d\left(A1,A2\right)$$

### 3.3. Classification

**Figure 7:**
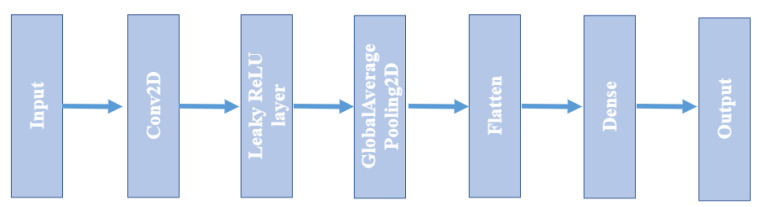
CNN2D model core.

After an encoding step, a classification step with the encoded data is important to test the efficiency of the proposed encoding technique. In this work, a CNN2D structure is used as a classifier, as illustrated in Figure 7.

After one 2D convolution block, features are fed into a global average pooling (GAP) layer [6]. GAP is a pooling operation designed to replace the fully connected layers in classical CNNs. The idea is to generate one feature map for each corresponding category of the classification task. The resulting vector is fed directly into the softmax layer to realize the final classification. The advantage of GAP regarding all over connected layers is that it is more local to the convolution structure, i.e., it enforces the correspondences between the feature maps and categories. Hence, the feature maps can be interpreted easily as category certainty maps. A second advantage is that there is no parameter optimization in the GAP. So, over fitting is kept away at this layer. In addition, GAP aggregates out the spatial information, and consequently it is more robust to the input spatial translations.

## 4. Case Study

To test the efficiency of the proposed framework, a two-year dataset from 4th November 2010 to 30th June 2011 is used for testing.

### 4.1. Test Bed and Data Set Description

**Figure 8:**
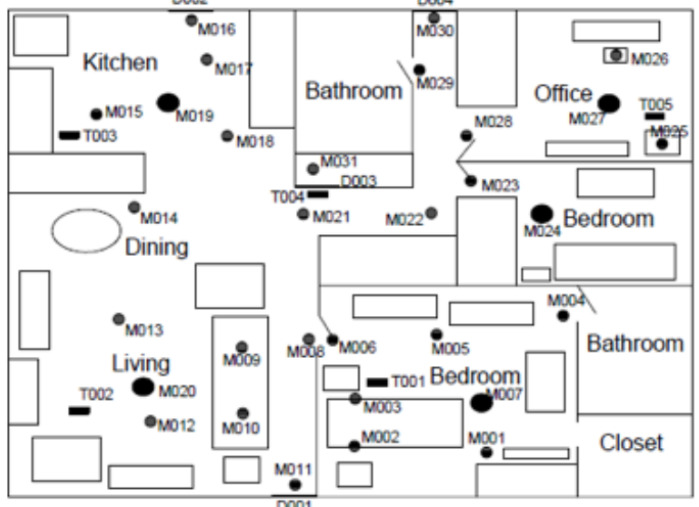
Aruba test bed.

The case study is performed on Aruba dataset which contains human activities collected in a smart apartment by the Center for Advanced Studies in Adaptive Systems (CASAS) [13]. The apartment accommodates a woman adult and has frequent visits from the woman’s children and grandchildren throughout the year. The configuration of sensors in the apartment is shown in Figure 8.

The Aruba dataset is different from numerous other public available datasets in terms of:

- Datasets relabeling—although the Aruba dataset originally included numerous activities (11 daily activities) in a long period of time.
- Anomaly detection and cleaning—after a detailed analysis of the existing public datasets, we noticed that they can contain anomalies. In fact, they may contain duplicated data, full or partial days. Some rows in the file contains character errors, e.g., “Oc” instead of “ON” value. Also, sensor activation traces in the dataset are not correctly ordered temporarily, i.e., within the time series, events with a later timestamp may be recorded before events with an earlier timestamp. In the dataset, activities are labeled with a “begin” or “end” keyword to set the beginning and the end of activity. However, activities can be nested (i.e., begin1 begin2 end2 end1). Also, it appears that they are interleaved (i.e., begin1 begin2 end1 end2). According to these observations, it is therefore necessary to take into account these particular cases when pre-segmenting the dataset into sequences of activities.

The set-up for the sensor network includes 5 temperature sensors, 3 door contact sensors, and 31 detection motion sensors. The sensors’ identifiers begin respectively with “T”, “D”, and “M”. In this work, a minimal set-up of sensors is considered in the case study. It includes 11 sensors which are the following: M003, M004, M009, M013, M014, M015, M017, M020, M026, M030, and D004.

The data are sampled with a sampling time equal to 1 s. This value is justified by the fact that if we increase the sampling rate we lose information. So, the smaller the value is, the richer the segment is in events and the more accurate the segmentation will be.

**Figure 9:**
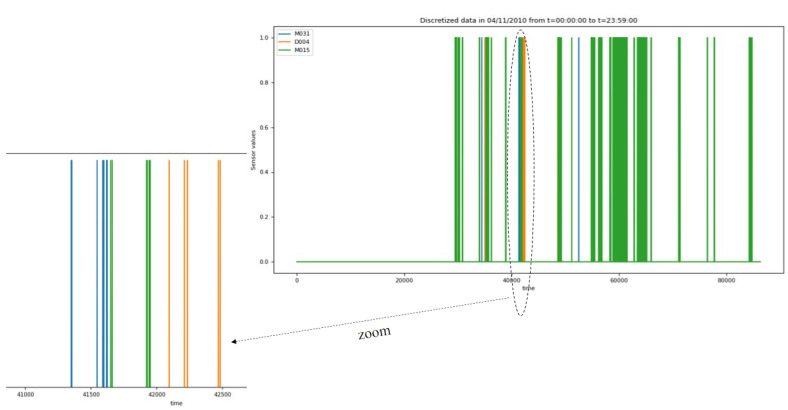
Sampled detection motions.

Figure 9 shows the time series of two motion detection sensors (M015 and M031) as well as the data of door contact sensor (D004) on 4 November 2010 from t = 00: 00: 00 to t = 23: 59: 00.

Table 1 summarizes the labeled activities in Aruba dataset.

### 4.2. Hypotheses and Key-words

In this work, several hypotheses are considered:

**Hypothesis** **1.** *Only binary sensors (detection motion and door contact sensors) are considered in this work, because the temperature sensors cannot deliver information regarding the location of occupant.*

**Hypothesis** **2.** *The following types of activities are concerned in this work: sleeping, bed to toilet, meal preparation, eating, work, housekeeping, relax, leave home, and enter home because they are the only labeled activities in our case study: the dataset Aruba.*

**Hypothesis** **3.** *In this work, we seek to discriminate activities pair by pair. For example, meal preparation and wash dishes which take place in the kitchen. The case of discrimination of three or more activities is not the subject of this study because in our case study, only two activities are overlapped. However, the case of discrimination of more than two activities must be treated two by two.*

### 4.3. Simulation Results

**Figure 10:**
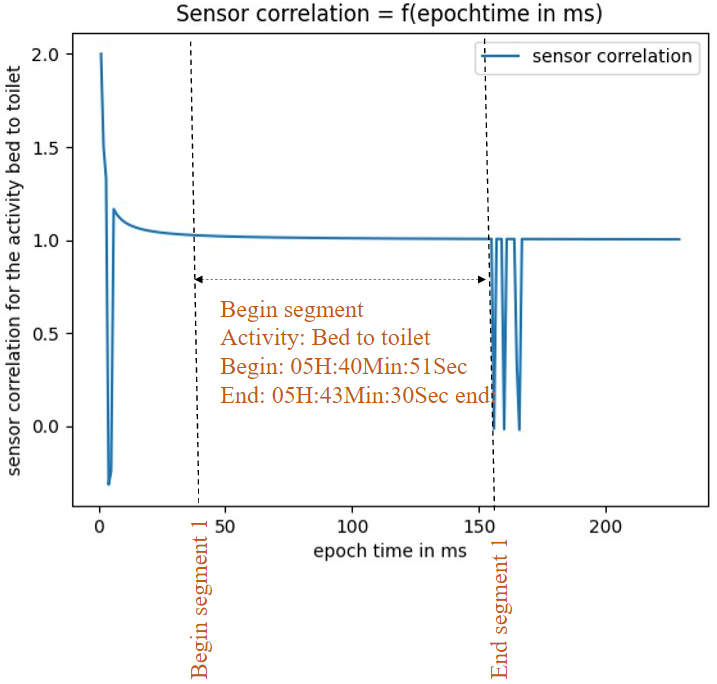
Corresponding segment of the activity “bed to toilet”.

**The first step** of the considered methodology is the real-time dynamic segmentation method, which is investigated in [35] and which adopts a dynamic window size. Figure 10 shows an example of real-time dynamic segmentation on 4th November 2010. The segment is defined by its beginning (5 h: 40 min: 51 s) and its end (5 h: 43 min: 30 s). Once the spacial event correlation is equal to 1 and the difference between the beginning and the end corresponds to the usual duration of the “bed to toilet” activity, we can conclude the corresponding segment of the activity “bed to toilet”.

An example of obtained segments using this approach on 4th November 2010 are given in Table 2.

**The second step** of the considered methodology is to encode each segment using the methodology described in Section 3.2. In the case of the Aruba dataset, only two activities (Meal Preparation and Wash Dishes) can overlap because they are realized in the same zone, which is the kitchen. The different tests carried out in this step do the following:

1. Calculate the representative matrix of each segment of activity for different values of parameter ρ using the concept of DWN. $M_{1}$ and $M_{2}$ represent, respectively, two matrices of two segments of activities “Meal Preparation” and “Wash Dishes” for ρ = 0.02. The periods of these segments, respectively, are the following:
  - Period 1: 4th November 2010 from 08: 11: 00 to 08: 27: 02 for meal preparation activity.
  - Period 2: 4th November 2010 from 10: 03: 21 to 10: 04: 25 for wash dishes activity.
  $$M_{1}=\left[\begin{array}{cccccccccc} 0 & 0 & 0 & 0 & 0 & 0 & 0 & 0 & 0 & 0\\ 0 & 0 & 0 & 0 & 0 & 0 & 0 & 0 & 0 & 0\\ 0 & 0 & 0 & 0 & 0 & 0 & 0 & 0 & 0 & 0\\ 0 & 0 & 0 & 0 & 0 & 0 & 0 & 0 & 0 & 0\\ 0 & 0 & 0 & 0 & 2.62 & 0 & 0 & 0 & 0 & 0\\ 0 & 0 & 0 & 0 & 0 & 0 & 0 & 0 & 0 & 0\\ 0 & 0 & 0 & 0 & 0 & 0 & 0 & 0 & 0 & 0\\ 0 & 0 & 0 & 0 & 0 & 0 & 0 & 0 & 0 & 0\\ 0 & 0 & 0 & 0 & 0 & 0 & 0 & 0 & 0 & 0\\ 0 & 0 & 0 & 0 & 0 & 0 & 0 & 0 & 0 & 0 \end{array}\right]M_{2}=\left[\begin{array}{cccccccccc} 0 & 0 & 0 & 0 & 0 & 0 & 0 & 0 & 0 & 0\\ 0 & 0 & 0 & 0 & 0 & 0 & 0 & 0 & 0 & 0\\ 0 & 0 & 0 & 0 & 0 & 0 & 0 & 0 & 0 & 0\\ 0 & 0 & 0 & 0 & 0 & 0 & 0 & 0 & 0 & 0\\ 0 & 0 & 0 & 0 & 0.97 & 0 & 0 & 0 & 0 & 0\\ 0 & 0 & 0 & 0 & 0 & 0 & 0 & 0 & 0 & 0\\ 0 & 0 & 0 & 0 & 0 & 0 & 0 & 0 & 0 & 0\\ 0 & 0 & 0 & 0 & 0 & 0 & 0 & 0 & 0 & 0\\ 0 & 0 & 0 & 0 & 0 & 0 & 0 & 0 & 0 & 0\\ 0 & 0 & 0 & 0 & 0 & 0 & 0 & -36 & 0 & 0 \end{array}\right]$$
  The Euclidean distance between $M2_{1}$ and $M_{2}$ is equal to 1.65.
2. Calculate the distance between the two matrix for each value of ρ.
3. Choose the value of ρ that maximizes the distance between two matrices. This value is calculated using Equation (3):

(3) $$\rho =ArgMax_{\rho_{i}}\left(d\left(M1,M2\right)\right)$$

Table 3 summarizes the Euclidean distance between the representative matrix for meal preparation activity and the representative matrix of wash dishes activity for different values of ρ.

To conclude, the optimal value of ρ is 0.02. This value is adopted to calculate the DWN for all activities in the dataset.

**The third step** of the considered methodology is the classification using a classifier. We use the CNN2D architecture to learn the activity classification model because it is adequate to the encoded segments of activities.

**Figure 11:**
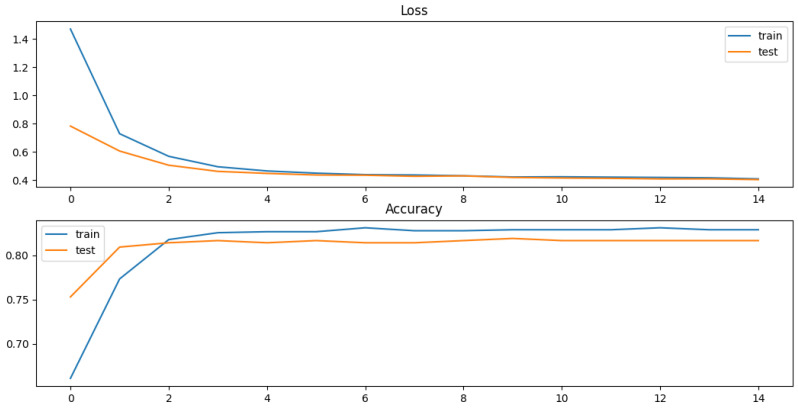
Evolution of accuracy and loss during training and validation phases.

A period of data from the year 2010 is used. Of this data, 70% is used for training the algorithm and 30% is used for testing. Figure 11 shows the evolution of accuracy and loss during training and validation phases.

In the training phase, the training accuracy and training loss are equal to 0.832 and 0.408, respectively. In the test phase, the test accuracy and the test loss are equal to 0.816 and 0.403, respectively .

The proposed framework for online HAR is compared with diverse available methods:

1. **Method 1: Time interval method**. In order to divide the sequence of sensor events into a set of segments, this method uses equal time intervals as input [34]. The resulting accuracy of classification using this method is equal to 0.774.
2. **Method 2: Sensor event based windowing**. This method is also investigated in [34]. In this method, each event contributes equally in the vector of features. The resulting accuracy of classification using this method is equal to 0.775
3. **Method 3: Sensor window–time window**. This method is also investigated in [34]. This method used a time-based weighting factor to calculate the contributions of each event regarding the feature vector. In this technique, there are an equal number of events in each window. The resulting accuracy of classification using this method is 0.78.
4. **Method 4: Sensor window–mutual information (SWMI)**. This method is also investigated in [34]. It assumes that the size of windows is constant and each window contains an equal number of sensor events. The extraction of features is based on the concept of mutual information, which is defined as the probability of the fact that these two sensor events appear sequentially in the entire dataset. The resulting accuracy of classification using this method is 0.783.
5. **Method 5: Incremental SVM algorithms**. This method is investigated in [37]. Two new methods for feature extraction that can be used for incremental learning HAR task are proposed.
  - Modified dependency sensor (MDS) feature extraction method. The dependency between two sensors depends on the triggering order of sensors in the dataset. It is computed by calculating their frequency of occurrence within an interval of n sensor events along the entire data sequence (instead of tracking, consecutive occurrences only when a specific activity runs).
  - Last-state (LS) sensor-based method. Considering the fact that segments of activities can contain sensors with active and inactive status (ON and OFF), sensors with high sensitivity can be triggered even if the occupant is not in the zone of these sensors. In this method, the authors made the hypothesis that the last-state of a sensor within a segment $Seg_{i}$ can be more informative and descriptive for the last event $E_{i}$. In this method, the feature vector is computed as follows: For each sensor $S_{i}$, if its last state within $Seg_{i}$ is [ON/OFF] then it will be represented by 1/−1, respectively, in the feature vector $F_{i}$, otherwise it will be represented by 0 (i.e., absent). The evaluation of the results obtained by these two methods of feature extraction gives an F-score of 0.658 using MDS and 0.67 using LS method.
6. **Method 6: Dynamic segmentation proposed by** [34]. The performance of this method is evaluated by an F-score of 0.607.
7. **Method 7: Liciotti model** [5]. The authors in [5] studied the performance of Liciotti algorithm for the HAR task. The given F-scores are respectively equal to 0.89 but the study is not carried out in real-time.

Table 4 summarizes these results of comparison. These results of comparison demonstrate that the proposed framework obtains ameliorated performance compared with other ordinarily used models. The reasons for this follow.

Firstly, insomuch as the segmentation of window is concerned, the sensor events in a segmented window should match to the aim event in space and time to provide an accurate context for the latest event as much as possible. The real-time dynamic segmentation approach considers the spatio-temporal correlation of events in the sliding window and ensures that events from very different geographic zones in the building, or with a large interval of time, will not be placed in the same window.

Secondly, the stigmergic tracks obtained using the encoding technique can reflect the duration of residents staying at each position and approximately characterize the motion process. Additionally, the impacts of past sensor events on the context definition about the event of interest can be disabled by employing volatility.

## 5. Conclusions and Future Works

Recognizing human activities is important for many services in smart building. This paper proposed a framework of real-time HAR based on three steps: (1) real-time segmentation of sensor events using the method presented in [35]; (2) encoding segments into a multidimensional format using the concept of DWN under constraints of non overlapping activities; and (3) classification of the activities using a CNN2D that takes into inputs the DWN of each related activity segment.

**The first step** is to transform the sensor events into a set of segments. In this work, we investigate the dynamic segmentation presented in [35]. The method incorporates dynamic event segmentation based on spatial correlation calculated by the PMC coefficient and the knowledge of trigger sensor of activity to predict activities when new events are recorded. The goal is to determine whether the last triggered sensor event is an indication of a new activity, or if it belongs to the current activity. For this, the spacial correlation between sensor events in view of what can be observed in the history of past events is considered.

After segmentation (i.e., the determination of the beginning and end of each segment of activity), there is a need to classify the activity using a neural network classifier. The CNN2D plays the role of a slice-wise feature extractor that selects the most effective features from input data. However, it requires a multidimensional format in input and an activity is not carried out in the same way by an occupant. Therefore, it is necessary to encode the activity segment to a multidimensional format taking into account the human spatio-temporal tracks.

**The second step** is to design an encoder architecture for CNN for HAR in smart home. The encoder architecture adopts the DWN that draws the human trajectory tracks. The technique is inspired by the algorithms of ants. The DWN is calculated under constraint of non overlapping activities. The resulting output of the DWN is a matrix that serves in future works as input for the CNN.

**The third step** is classification using a CNN2D classifier architecture. The methodology was validated on Aruba dataset. The performance of the proposed approach is demonstrated to be better than that of existing methods in comparative experiments with an accuracy of 81.6%. Consequently, the effectiveness of the proposed online activity recognition method integrating the dynamic segmentation and emergent modeling is proven. In addition, experiments were conducted to measure the computation time of our proposed framework (segmentation, encoding, and classification with a CNN2D architecture) and the computation time using Liciotti-based LSTM classification algorithm [38] under the same conditions (i.e., same dataset, experiment on the same benchmark with the same characteristics). In the first case, the calculation time is equal to 23.48 s and it is of the order of 1 min for the second case. Therefore, the proposed framework is more efficient in terms of computation time, with a performance of 39.13%. This makes us think that the implementation of an embedded system can be as efficient in terms of computation time.

Future works will address the following:

- Testing the efficiency of this method on different hardware architectures considering the cost of each solution.
- In a “digital-twin” approach, use a simulator to surmount the problem of obtaining real data, generate synthetic data to introduce the different variations (different environment, user habits, and different sensors) according to a real-time use case.

## Figures and Tables

**Table 1:** Annotated activities in Aruba dataset.

Activity Name	Number of Activities
Meal Preparation	1606
Relax	2910
Eating	257
Work	171
Sleeping	401
Bed to Toilet	157
Enter Home	431
Leave Home	431
Housekeeping	33

**Table 2:** Results of segmentation.

Activity	Ground Truth Segment	Simulated Segment
Sleeping (1)	begin: 0 h: 3 min: 50 s	begin: 0 h: 3 min: 50 s
	end: 5 h: 40 min: 43 s	end: 5 h: 40 min: 44 s
Bed to Toilet	begin: 5 h: 40 min: 51 s	begin: 5 h: 40 min: 51 s
	end: 5 h: 43 min: 30 s	end: 5 h: 43 min: 24 s
Sleeping (2)	begin: 5 h: 43 min: 45 s	begin: 5 h: 43 min: 45 s
	end: 8 h: 1 min: 12 s	end: 8 h: 1 min: 9 s
Meal Preparation	begin: 8 h: 11 min: 9 s	begin 8 h: 11 min: 15 s
	end: 8 h: 27 min: 2 s	end: 8 h: 24 min: 48 s

**Table 3:** Distance between the two representative activity matrix.

ρ	0.02	0.09	0.2	0.5
$d(M_{1},M_{2})$	1.65	0.69	0.17	0

**Table 4:** Comparison results of classification using different neural network architectures and different segmentation methods.

Method	Accuracy
Proposed method	0.816
Time interval method	0.774
Sensor event based windowing	0.775
Sensor window–time window	0.780
SWMI	0.783
DS	0.607
MDS	0.658
LS	0.67
Liciotti	0.89

## Data Availability

Samples of the compounds are available from the authors.

## Abbreviations

The following abbreviations are used in this manuscript:

HAR	Human Activity Recognition
CNN	Convolutional Neural Network
DWN	Directed Weighted Network
FTW	Fuzzy Time Interval
SC	Sensor Correlation
LSTM	Long Short Time Memory
SEW	Sensor Event Window
PMC	Pearson product moment correlation
HMM	Hidden Markov Model
DDA	Data Driven approach
KDA	Knowledge-Driven Approache
